# HCAP Factor Structure: Results From The Northern Ireland Cohort For The Longitudinal Study Of Ageing

**DOI:** 10.1093/geroni/igaf122.1774

**Published:** 2025-12-31

**Authors:** Nicola Ann Ward, Calum Marr, Leeanne O’Hara, Charlotte Neville, Michael McAlinden, Claire Potter, David R Weir, Bernadette McGuinness

**Affiliations:** Queen’s University Belfast, Belfast, Northern Ireland, United Kingdom; Queen’s University Belfast, Belfast, Northern Ireland, United Kingdom; Queen’s University Belfast, Belfast, Northern Ireland, United Kingdom; Queen’s University Belfast, Belfast, Northern Ireland, United Kingdom; Queen’s University Belfast, Belfast, Northern Ireland, United Kingdom; Queen’s University Belfast, Belfast, Northern Ireland, United Kingdom; University of Michigan, Ann Arbor, Michigan, United States; Queen’s University Belfast, Belfast, Northern Ireland, United Kingdom

## Abstract

Dementia affects nearly one million people in the United Kingdom, with Northern Ireland (NI) projected to experience the highest increase due to rapid population aging. Accurate prevalence estimates are essential for public health planning and predicting future societal and economic needs. The Harmonized Cognitive Assessment Protocol (HCAP) enables standardized cross-country comparisons of dementia prevalence. HCAP was implemented as a substudy of the NI Cohort for the Longitudinal Study of Aging (NICOLA). The current study aimed to validate the cognitive factor structure of HCAP, previously implemented in HRS-HCAP, in a representative Northern Irish population using confirmatory factor analysis (CFA). The NICOLA-HCAP subsample included 1,037 randomly selected community-dwelling adults aged ≥65 who completed neurocognitive tests. CFA was conducted in STATA(v15.1) to evaluate single and multi-domain factor structures. All factor scores were presented as mean ± SD. A preliminary five-factor model encompassing memory (MEM: -0.16 ± 0.82), executive functioning (EXF: -0.15 ± 0.64), visuospatial (VIS: -0.02 ± 0.11), orientation (ORI: -0.01 ± 0.05), and language (LAN: -0.10 ± 0.43), with a higher-order general cognition factor (GCOG: -0.15 ± 0.64), was identified (SRMR = 0.043, RMSEA = 0.055, CFI = 0.932). GCOG strongly influenced MEM, EXF, and ORI (loadings >0.80) and had moderate associations with VIS (0.70) and LAN (0.67). The model fits well, aligning with other HCAP models. Next steps will involve selecting the normative HCAP sample, standardizing scores by age and gender, and establishing diagnostic cutoffs for impairment (>1.5SD below the mean in two domains derived from the model with informant-rated deficits).

